# Transcriptome Analysis of Porphyrin-Accumulated and X-Ray-Irradiated Cell Cultures under Limited Proliferation and Non-Lethal Conditions

**DOI:** 10.3390/microarrays4010025

**Published:** 2015-01-27

**Authors:** Junko Takahashi, Masaki Misawa, Hitoshi Iwahashi

**Affiliations:** 1 Biomedical Research Institute, National Institute of Advanced Industrial Science and Technology, Tsukuba, Ibaraki 305-8566, Japan; 2 Human Technology Research Institute, National Institute of Advanced Industrial Science and Technology, Tsukuba, Ibaraki 305-8564, Japan; E-Mail: m.misawa@aist.go.jp; 3 Faculty of Applied Biological Sciences, Gifu University, Gifu, Gifu 501-1193, Japan; E-Mail: h1884@gifu-u.ac.jp

**Keywords:** radiotherapy, X-ray, sensitizer, photodynamic therapy, 5-aminolevulinic acid (ALA), protoporphyrin IX (PpIX), cancer, microarray

## Abstract

5-Aminolevulinic acid (ALA) is a precursor of the photosensitizer used in photodynamic therapy. It accumulates in tumor cells and subsequently metabolizes to protoporphyrin IX (PpIX), which generates singlet oxygen after light irradiation. PpIX enhances the generation of reactive oxygen species following physicochemical interactions with X-rays. ALA-based treatment using fractionated doses of irradiation suppressed tumor growth in a mouse melanoma model. To study the transcriptomic effects of PpIX, microarray analyses were conducted using HeLa cells with limited proliferation capacity. Based on the *p*-values (*p* < 0.01), we selected genes showing altered expression in each treatment group with reference to the non-treatment (NT) group. We detected 290, 196 and 28 upregulated genes, as well as 203, 146 and 36 downregulated genes after a 6 h-long PpIX treatment (1 μg/mL) prior to 3 Gy X-ray irradiation (PpIX-XT), 3 Gy X-ray irradiation alone (XT) and PpIX treatment alone (PpIXT), respectively. Functional analysis revealed that a majority of the regulated genes in the XT and PpIX-XT groups were related to cell-cycle arrest. The XT and PpIX-XT groups differed in the quantity, but not in the quality of their gene expression. The combined effect of PpIX and X-ray irradiation sensitized HeLa cells to X-ray treatment.

## 1. Introduction

Photodynamic therapy (PDT) is used to treat certain cancerous and pre-cancerous dermatological conditions. It is preferred over surgical resection, because, just like radiotherapy, it is a non-invasive procedure. PDT was established in the 1970s, and it is based on the interaction of light with photosensitive agents, known as photosensitizers. These molecules preferentially accumulate in target cells and initiate energy transfer, as well as local chemical effects [1]. After exposure to specific wavelengths of light, the photosensitizer is excited from its ground state to its singlet state. It subsequently undergoes Type I (electron transfer) and/or Type II (energy transfer) reactions to produce reactive oxygen species (ROS), resulting in necrosis and/or apoptosis of the exposed cells [2]. The successful use of PDT requires photoactivation of the corresponding photosensitizers. The low penetration depth of incident light through tissues limits treatment with PDT to tumors located on, or right below, the skin surface or to the lining of some of the internal organs. In our previous study, protoporphyrin IX (PpIX) was examined as a candidate PDT photosensitizer with biological compatibility. We examined the type and quantity of ROS species generated by X-ray irradiation [3]. Solutions containing ROS-detecting reagents (aminophenyl fluorescein (APF) and dihydroethidium (DHE)), an ethanol quencher, and different concentrations of PpIX were used to estimate the contribution of PpIX in the generation of the hydroxyl radical (∙OH), the superoxide anion (O_2_^−^) and the singlet oxygen (^1^O_2_) species. 

Currently, 5-aminolevulinic acid (ALA)-PDT is used to treat a variety of neoplastic and non-neoplastic conditions, because its phototoxicity is weaker than that of porphyrin derivatives. This is because ALA is not directly activated by light, unlike porphyrin derivatives [4]. ALA, a heme precursor and pro-drug, is absorbed and converted by the heme biosynthetic pathway to photoactive PpIX. After light exposure, PpIX accumulates preferentially in rapidly dividing cells, without showing any photo-induced cytotoxicity. ALA’s pharmacokinetics, toxicity and accumulation in cancer cells are well understood. Furthermore, ALA has been approved for use as a precursor of the natural photosensitizer, PpIX, in PDT in the U.S. and in Europe [5]. We also investigated the effect of ALA pre-treatment (prior to X-ray irradiation) on mouse B16-BL6 melanoma cells *in vitro* and *in vivo*. ALA enhanced the accumulation of PpIX in tumor cells and increased X-ray-induced ROS generation *in vitro*. Furthermore, the administration of ALA prior to irradiation improved tumor suppression in animals undergoing fractionated irradiation [6]. 

To evaluate the effects of PpIX during X-ray treatment on the cellular regulation of transcription, we performed a microarray analysis. HeLa cells were used as the typical cell type for human cancers. To evaluate the use of radio-sensitizers in radiotherapy, we selected non-lethal cells with limited proliferation capacity for the microarray analysis. To assess the influence of the post-initial response, we carried out expression analysis 24 h after X-ray irradiation. The present study employed microarray analysis to quantify genes that are upregulated or downregulated as a result of PpIX treatment prior to X-ray irradiation (PpIX-XT), X-ray irradiation alone (XT) and PpIX treatment alone (PpIXT), as determined by comparing treated cells to those of the non-treatment (NT) group. Genes were classified according to their biological functions and properties. Our results characterized treatment-related gene expression changes resulting from PpIX administration, thus identifying the molecular basis of its radio-sensitizing effects. 

## 2. Experimental Section

Chemicals: PpIX was purchased from Sigma (St. Louis, MO, SUA). The cell proliferation reagent 4-[3-(4-iodophenyl)-2-(4-nitrophenyl)-2H-5-tetrazolio]-1,3-benzene disulfonate (WST-1) was purchased from Roche (Mannheim, Germany). APF was purchased from Sekisui Medical Co. Ltd. (Tokyo, Japan). DHE was purchased from Life Technologies (Tokyo, Japan). Methanol, perchloric acid, acetic acid, sodium hydrogen carbonate, DMEM medium, penicillin, streptomycin, fetal bovine serum and PBS were purchased from Wako Chemicals (Osaka, Japan).

Cell culture: The HeLa cell line was supplied by the Riken Cell Bank (Tsukuba, Japan). The cells were cultured in DMEM containing 10% FBS in a 5% CO_2_ humidified incubator at 37 °C. The medium was supplemented with 100 units/mL penicillin and 100 μg/mL streptomycin. 

X-ray irradiation: HeLa cells were irradiated (0, 1, 3 or 5 Gy) using 160-kV X-rays (Faxitron^®^Cabinet X-ray System Model CP-160, Wheeling, IL, USA). A Unidos^®^ E Universal Dosimeter (PTW, Freiburg, Germany) was used to measure the dose rate. The resulting dose rate was 1 Gy/min.

Determination of porphyrin concentration in cells: Intracellular porphyrin was isolated in 1 N perchloric acid and methanol (1:1 v/v) after homogenization and centrifugation at 3000 rpm for 10 min. The number of cells used to determine the porphyrin concentration was approximately 2.0 × 10^6^ cells. The supernatant was transferred to a tube, neutralized with sodium hydrogen carbonate and acidified with acetic acid. Porphyrin concentration was determined by spectrophotometry at the Soret maximum (405 nm) and by fluorescence, using an excitation wavelength of 405 nm and an emission wavelength of 630 nm [7]. 

Cell viability assay: The WST-1 cell viability assay was performed to assess cellular responses to PpIX treatment and X-ray irradiation. WST-1 was used as described by the manufacturer. As the reagent is based on the cleavage of the tetrazolium salt, WST-1, by mitochondrial dehydrogenases in viable cells, the absorbance of the formazan dye is supposed to correlate with the number of active cells in the medium. Before X-ray exposure, HeLa cells were cultivated in 96-well plates to confluency. PpIX was added 6 h before X-ray irradiation in 100 μL of culture medium at concentrations of 0, 0.3, 1 and 3 μg/mL. The plates were irradiated at a dose of 0, 1, 3 and 5 Gy. Immediately after irradiation, the medium was removed, and 100 μL of fresh medium was added. 1, 24 and 72 h after irradiation, and 10 μL WST-1 was added to each well. The plates were incubated in a 5.0% CO_2_ humidified incubator at 37 °C for 1 h, following which, the absorbance at 450 nm was measured using a plate reader against a referenced absorbance at 600 nm. Relative cell viability was defined as the dye absorption ratio of treated *versus* untreated cells. 

Clonogenic ability: Clonogenic survival ability was detected by a colony-forming assay based on the methods of Franken *et al.* [8]. Briefly, cells were seeded in six-well microplates at a density of 500 cells per well. Cells were allowed to attach for 14 h and treated with PpIX and/or X-ray. PpIX was added 6 h before X-ray irradiation in 3 mL of culture medium at concentrations of 0, 0.3, 1 and 3 μg/mL. The plates were irradiated at a dose of 0, 1, 3 and 5 Gy. After X-ray irradiation, cells were washed with PBS and replaced into fresh medium. Cells were then cultured for the period required for control cells to form colonies (one colony was defined as a colony containing 50 or more cells) or for 7 days. The experiments were performed on plates kept in the dark to avoid the effects of light. After fixation with 100% methanol for 15 min, cells were stained with Giemsa solution (diluted 1:50 in water) for 15 min and rinsed with distilled water. The number of colonies was counted.

Measurement of intracellular ROS: HeLa cells were cultivated in 96-well plates up to confluency. PpIX was added 6 h before X-ray irradiation in 100 μL of culture medium at concentrations of 0, 0.3, 1 and 3 μg/mL. After washing with PBS, APF or DHE was added with fresh medium at a final concentration of 10 μM. The plates were incubated in the dark for 30 min at 37 °C and irradiated at a dose of 0, 1, 3 and 5 Gy. After irradiation, the medium was removed, and the cells were washed with PBS. Fluorescence was measured on a microplate reader (Infinite M200, TECAN) at excitation/emission and λ = 490 nm/515 nm for APF and at excitation/emission and λ = 510 nm/580 nm for DHE. 

Minimum information about a microarray experiment (MIAME) compliance and data availability: The microarray experiments described in this manuscript are MIAME compliant, and the raw data have been deposited in the Gene Expression Omnibus (GEO) database (Accession Number GSE 61805) [9]. 

Sample preparation and microarray assays: RNA was extracted from cells using the Qiagen RNeasy Mini kit (Qiagen GmbH, Germany), according to the manufacturer’s guidelines. The quality of the purified RNA was verified using an Agilent^®^ 2100 Bioanalyzer (Agilent Technologies, Santa Clara, CA, USA). RNA concentration was determined using a NanoDrop^®^ ND-1000 spectrophotometer (NanoDrop Technologies, Wilmington, DE, USA). Fluorescent cyanine 3-cytidine triphosphate (CTP)-labeled cRNA was used for hybridization to human oligo microarray slides (#G4112F, Agilent Technologies) at 65 °C for 17 h. The hybridized microarray slides were washed according to the manufacturer’s instructions and were scanned with an Agilent DNA Microarray Scanner (#G2565BA, Agilent Technologies) at a resolution of 5 μm. The scanned images were analyzed quantitatively using Agilent Feature Extraction Software version 9.5.3.1 (Agilent Technologies).

Microarray data analysis: Data were normalized by quantile normalization and were analyzed using GeneSpring GX software version 10.0.1 (Agilent Technologies). The Gene Ontology (GO) Database (http://www.geneontology.org/) was used to functionally categorize gene-expression profiles. GO terms were obtained from Agilent Technologies eArray [10]. Microarray cDNA probes were classified according to GO terms for different biological processes.

Statistical analysis: Accumulation of porphyrins in HeLa cells was analyzed by a two-tailed Student’s *t*-test. Data for cell viability, colony-forming ability and intracellular ROS were analyzed by one-way analysis of variance (ANOVA). The Tukey–Kramer HSD test was used for *post hoc* pairwise comparison. Differences were considered statistically significant at *p* < 0.01. 

The Student’s *t*-test was used to assess the significance of each gene in the microarray. We selected genes in the PpIXT, XT and PpIX-XT groups with *p*-values < 0.01 when compared to the NT group. After excluding overlapping probes, genes with significantly different expression in each group (PpIXT, XT and PpIX-XT) were analyzed using the functional annotation chart in the Database for Annotation, Visualization and Integrated Discovery (DAVID Bioinformatic Resources 2007, National Institute of Allergy and Infectious Disease) [11,12,13]. To characterize gene expression, the Fisher’s exact test was applied to calculate the significance for GO terms related to biological processes. Terms with adjusted *p*-values < 0.01 (Benjamini–Hochberg FDR correction) were defined as statistically significant.

## 3. Results and Discussion

### 3.1. PpIX Uptake by HeLa Cells

**Figure 1:**
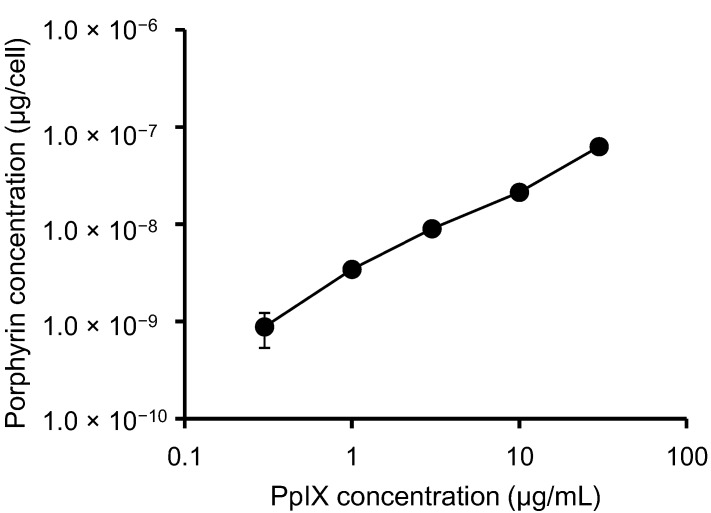
Total porphyrin levels in HeLa cells. Cells were incubated with different concentrations of PpIX for 6 h. The data shown correspond to the mean ± SD (n = 4).

To study the behavior of PpIX in HeLa cells, we examined intracellular porphyrin levels. During porphyrin-mediated PDT, PpIX is absorbed into tumor cells, where it them accumulates [14]. While other porphyrins are detected using this method, PpIX is the major porphyrin found in cells and tissues [15]. HeLa cells were incubated with increasing concentrations of PpIX for 6 h, and the levels of porphyrin uptake were measured (Figure 1). We found that PpIX was rapidly taken up by the cells, and intracellular porphyrin accumulation increased with increasing PpIX concentrations.

### 3.2. Determination of Cell Viability and Clonogenic Survival in Irradiated, PpIX-Treated HeLa Cells

**Figure 2:**
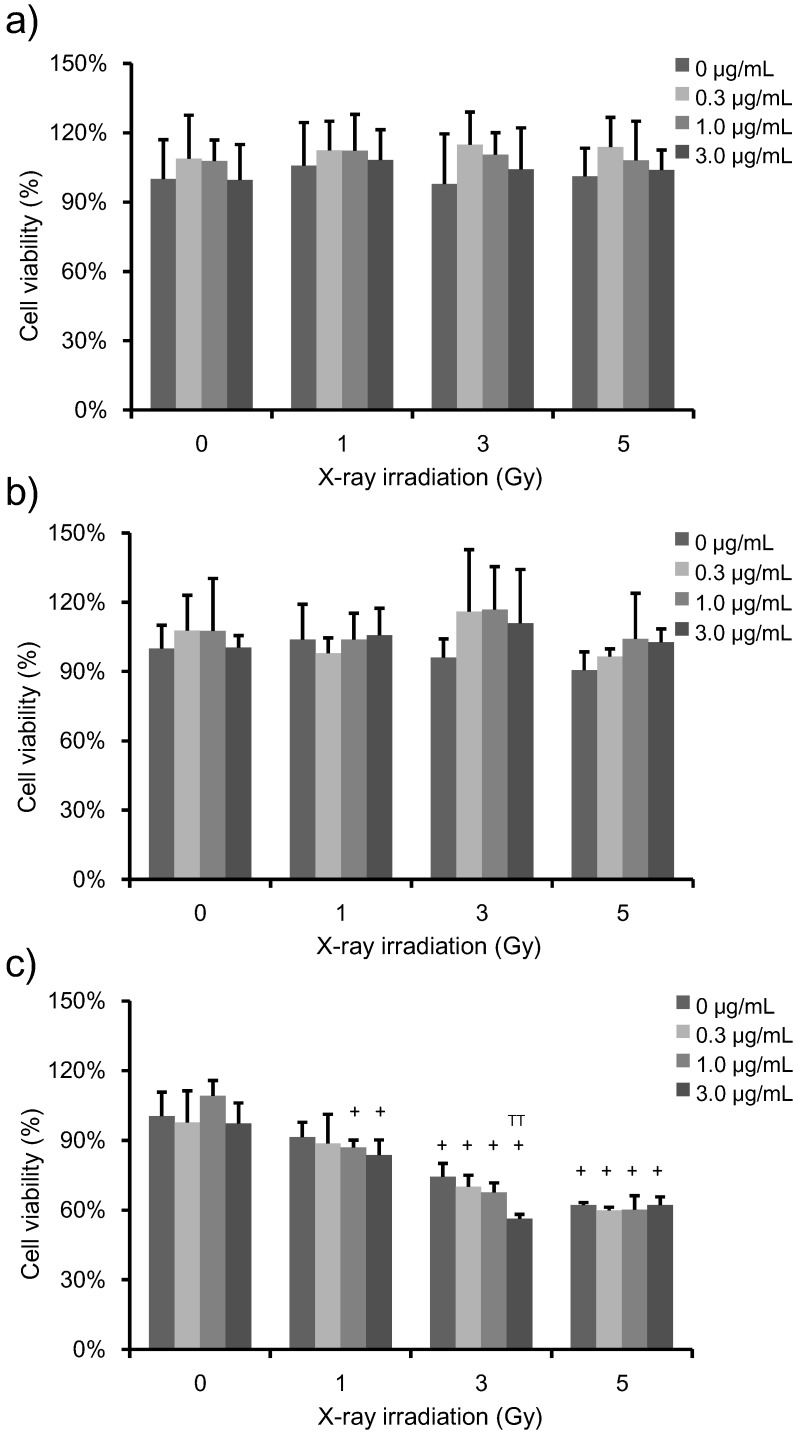
Viability of HeLa cells treated with increasing PpIX concentration and X-ray exposure. The data correspond to the mean ± SD at 1 h post-irradiation (**a**) (n = 6), at 24 h post-irradiation (**b**) (n = 4) and 72 h post-irradiation (**c**) (n = 4). The cell proliferation reagent 4-[3-(4-iodophenyl)-2-(4-nitrophenyl)-2H-5-tetrazolio]-1,3-benzene disulfonate (WST-1) was used to quantify cell viability. The percent survival is shown relative to untreated control cells. No change was observed in cell viability upon increasing the X-ray dose and PpIX concentration in control cells. Statistical significance (*p* < 0.01) relative to the experiment performed without PpIX treatment at the same irradiation dose is indicated by (^TT^). Statistical significance (*p* < 0.01) relative to the experiment performed without irradiation at the same PpIX concentration is indicated by (^+^).

To determine the effects of PpIX + X-ray treatments on HeLa cell viability, we performed a WST-1 assay (Figure 2). HeLa cells were incubated with increasing concentrations of PpIX for 6 h prior to irradiation. PpIX-treated cells were subjected to increasing levels of X-ray exposure, and then, cell viability was analyzed at 1 h, 24 h and 72 h post-irradiation. The percent of survival was expressed with reference to non-irradiated control cells. No change in cell viability was observed with increasing X-ray doses in the absence of PpIX. Furthermore, no change in cell viability was observed by increasing PpIX concentration at any of the X-ray doses at 1 h and 24 h post-irradiation (Figure 4a,b). However, at 72 h post-irradiation, cell viability decreased as the PpIX concentration and X-ray irradiation dose increased (Figure 4c).

**Figure 3:**
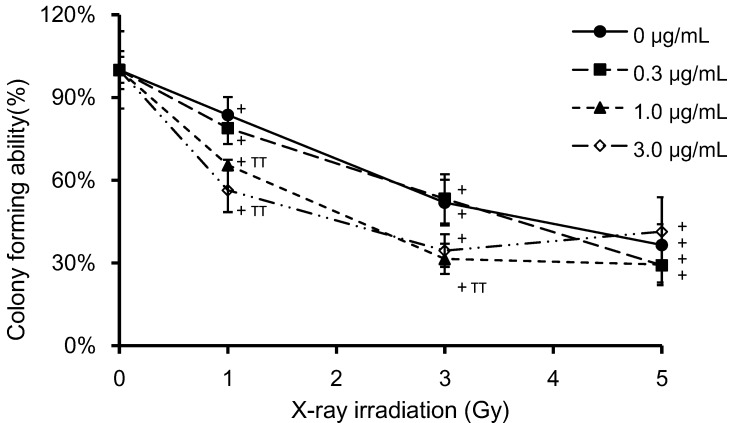
HeLa cell colony forming was measured by the clonogenic assay. Cells were grown in six-well plates and exposed to various concentrations of PpIX and doses of X-ray irradiation. After irradiation, the cell culture medium was removed, the cells were washed with PBS and fresh medium was added. Cells were then cultured for seven days, and the cell colony formation was quantified. Clonogenic survival was standardized to a non-irradiated control (100%) treated with the same PpIX concentration. The data correspond to the mean ± SD (n = 4). Statistical significance (*p* < 0.01) relative to the experiment performed without PpIX at the same irradiation dose is indicated by (^TT^). Statistical significance (*p* < 0.01) relative to the experiment performed without irradiation at the same PpIX concentration is indicated by (^+^).

Figure 3 shows the effects of PpIX on clonogenic survival at different X-ray doses. HeLa cells were incubated with PpIX for 6 h prior to irradiation. Clonogenic survival is expressed with reference to non-irradiated control cells at the same PpIX concentration. Clonogenic survival decreased with increasing X-ray doses and PpIX concentration. ANOVA revealed a significant difference (*p* < 0.01) in clonogenic survival between the non-irradiated and the 1, 3 and 5 Gy-irradiated samples at equivalent PpIX concentrations. A significant difference (*p* < 0.01) was also found between control cells (no PpIX) and cells treated with 1 μg/mL PpIX in the 1 and 3 Gy-irradiated samples, as well as between the control (no PpIX) and cells treated with 3 μg/mL PpIX in the 1 Gy-irradiated sample. 

Taken together, these results indicate that PpIX treatment does not affect cellular metabolic activity at 1 h and 24 h post-irradiation, but it does affects clonogenic cell survival. Thus, for the microarray analysis, we selected the condition corresponding to 3 Gy-irradiated cells. This radiation dose affected clonogenic survival, but did not affect cell viability after 24 h, when X-ray-irradiation was the only treatment involved. Moreover, to study the effect of PpIX at the same X-ray dose, we chose to use 1 μg/mL, as this dose affected clonogenic survival.

The effect of radiosensitization becomes noticeable when the effect of PpIX becomes relatively greater than the X-ray radiation damage alone. In our study, we consider that the contribution of PpIX to the cell viability and the clonogenic survival was dominant at 1 and 3 Gy, but the X-ray radiation damage was exceedingly greater than the radiosensitization at 5 Gy.

### 3.3. Generation of ROS by PpIX and X-Ray Irradiation

**Figure 4:**
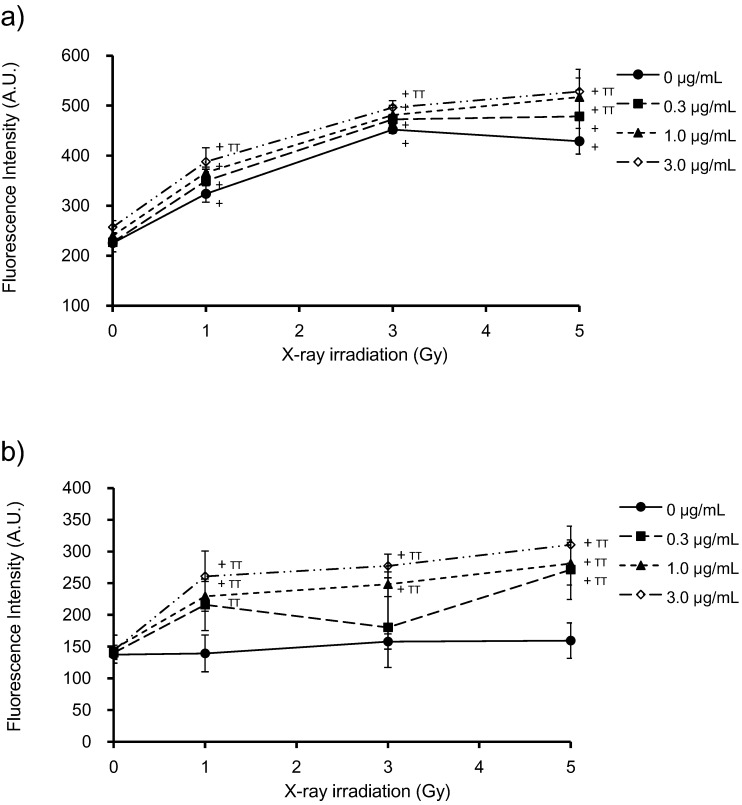
Detection of intracellular ROS levels in HeLa cells exposed to increasing PpIX concentrations and X-ray doses, using aminophenyl fluorescein (APF) (**a**) or dihydroethidium (DHE) (**b**). PpIX was added 6 h prior to X-ray irradiation. Before X-ray irradiation, APF or DHE was added to HeLa cells at a final concentration of 10 μM and incubated in the dark for 30 min at 37 °C. After X-ray irradiation, the medium was removed, and the cells were washed with PBS. The resulting fluorescence was measured using a microplate reader. The data correspond to the mean ± SD (n = 4). Statistical significance (*p* < 0.01) relative to the experiment performed without PpIX at the same irradiation dose is indicated by (^TT^). Statistical significance (*p* < 0.01) relative to the experiment performed without irradiation at the same PpIX concentration is indicated by (^+^).

Intracellular ROS production was assessed by pre-incubation of HeLa cells with cell-permeable fluorescent probes (APF or DHE) that show different sensitivities to specific ROS species (∙OH, O_2_^−^ and ^1^O_2_). HeLa cells were incubated with PpIX for 6 h prior to X-ray irradiation. Cells without PpIX treatment were irradiated under the same conditions as those used for controls. ROS levels are expressed in terms of fluorescence intensity. Figure 4a shows the effects of PpIX on intracellular ROS levels at different X-ray doses in the presence of APF. X-ray irradiation in the absence of PpIX increased ROS levels in HeLa cells. After pre-incubation with PpIX, ROS levels increased with the X-ray dose and PpIX concentration. Figure 4b shows the effects of PpIX on the intracellular ROS levels for various X-ray doses in the presence of DHE. No change was observed in the intracellular ROS by increasing X-ray irradiation in the absence of PpIX. After pre-incubation with PpIX, the ROS levels increased with PpIX concentration and X-ray dose.

In our previous study, using standard reactions, we showed that APF had a higher sensitivity for detecting ∙OH, unlike DHE, which showed higher sensitivity for detecting O_2_^−^ and s^1^O_2_ [3]. Therefore, these results suggest that X-rays increase ∙OH generation and that PpIX enhances the production of ∙OH, O_2_^−^ and ^1^O_2_.

### 3.4. Microarray Gene Expression Analysis

For the microarray analyses, RNA was extracted from HeLa cells with no treatment (NT), cells treated with 1 μg/mL PpIX without X-ray irradiation (PpIXT), cells that had been irradiated with 3 Gy without PpIX treatment (XT) and cells that had been treated with both 1 μg/mL PpIX and 3 Gy-irradiation (PpIX-XT). In this experiment, the cells were collected 24 h after irradiation. These specific conditions were chosen, because X-ray exposure at a dose of 3 Gy did not affect cell survival 24 h post-irradiation, regardless of the concentration of PpIX treatment (Figure 2b). Furthermore, 1 μg/mL PpIX-treated cells were used because PpIX enhanced ROS generation induced by X-ray irradiation at this concentration (Figure 4b). Moreover, clonogenic survival was affected by 1 μg/mL PpIX treatment and X-ray irradiation (Figure 3). Transcriptional changes occurred as early as 0.5 h to 2 h after X-ray exposure and peaked shortly thereafter [16,17]. After that time, transcriptional changes were observed 12 to 24 h after irradiation [16,18,19]. In each group, the RNA sample was analyzed using a human gene expression microarray consisting of 43,376 oligonucleotide probes.

#### 3.4.1. Analysis of Microarray Gene Expression Profiles via the Classification of Upregulated and Downregulated Genes

We characterized the genes that were upregulated or downregulated by PpIX treatment prior to X-ray irradiation. Accordingly, we first selected genes with altered expression in each treatment group with respect to the NT group based on their *p*-values (*p* < 0.01). Then, these genes were classified as upregulated or downregulated after each treatment. In the PpIX-XT group, 290 genes met the selection criteria for upregulation, and 203 genes met the selection criteria for downregulation. In the XT group, 196 genes were upregulated and 146 genes were downregulated. In the PpIXT group, 28 genes were upregulated and 39 genes were downregulated (Table 1). The number of differentially-expressed genes was highest in the PpIX-XT group, followed by the XT and PpIXT groups. We selected genes for the subsequent functional analysis based on their *p*-value. This selection method was mathematically proven to consider both the fold, as well as the differences. 

**Table 1:** Summary of the microarray data with *p*-values < 0.01 in the PpIX treatment alone (PpIXT), 3 Gy X-ray irradiation alone (XT) and 3 Gy X-ray irradiation (PpIX-XT) groups, *versus* the non-treatment (NT) group.

Regulation	Number of genes		
	PpIXT	XT	PpIX-XT
Upregulation	28	196	290
Downregulation	39	146	203
Total	67	342	493

#### 3.4.2. Functional Analysis

The selected genes were analyzed using the functional annotation chart in the Database for Annotation, Visualization and Integrated Discovery (DAVID, version 6.7). Using this analysis, we extracted the GO terms based on the number of genes in each GO category. Table 2 shows the biological processes (as identified by the GO terms) that were differentially-represented with respect to the NT group (FDR < 0.01). Eighteen GO terms were identified among the upregulated genes in the PpIX-XT group, and six GO terms were identified among the downregulated genes. Twenty GO terms were identified among the upregulated genes in the XT group, and four GO terms were identified among the downregulated genes. Moreover, no GO terms were identified among the upregulated or downregulated genes in the PpIXT group. These GO terms corresponded to four functions: cell cycle regulation (15 GO terms), DNA metabolic processes (five GO terms), chromosome organization (10 GO terms) and cellular response to stress (two GO terms). The changes in gene expression after PpIX administration without X-ray irradiation (PpIXT) were less extensive than those in other treatment groups and were not systematic.

For each GO term corresponding to cell cycle regulation, the number of genes was higher and the *p*-value of the FDR was lower in the PpIX-XT group than in the XT group. These results indicated that cell cycle disturbances were induced in both the PpIX-XT and XT groups; however, the extent of these disturbances was higher in the PpIX-XT group than in the XT group. For a more detailed analysis of the molecular processes disrupted in XT and PpIX-XT groups, we next evaluated the genes that corresponded to the GO terms.

### 3.5. Functional Validation Using Marker Genes

Based on the results of the functional analysis, we examined the expression levels of genes involved in cell cycle regulation, DNA metabolic processes and chromosome organization. We selected genes with well-described functions in order to best characterize the corresponding biological responses.

**Table 2:** Biological processes based on GO terms that are differentially represented in the PpIXT, XT and PpIX-XT groups, with reference to the NT group. The data were generated using the functional annotation chart provided in Database for Annotation, Visualization and Integrated Discovery (DAVID).

Function	Accession	Term	Count			FDR			Gene list of upregulation or downregulation		
			PpIXT	XT	PpIX-XT	PpIXT	XT	PpIX-XT	PpIXT	XT	PpIX-XT
cell cycle	GO:0000075	cell cycle checkpoint			11			3.9 × 10^−4^			up
	GO:0000278	mitotic cell cycle		14	38		4.5 × 10^−3^	9.5 × 10^−20^		up	up
	GO:0007049	cell cycle			50			2.3 × 10^−18^			up
	GO:0007093	mitotic cell cycle checkpoint			8			1.8 × 10^−3^			up
	GO:0007346	regulation of mitotic cell cycle			15			1.4 × 10^−5^			up
	GO:0010564	regulation of cell cycle process			11			3.2 × 10^−3^			up
	GO:0022402	cell cycle process		18	44		1.0 × 10^−3^	9.2 × 10^−19^		up	up
	GO:0051726	regulation of cell cycle			22			2.4 × 10^−6^			up
	GO:0000280	nuclear division		12	28		9.2 × 10^−4^	5.6 × 10^−16^		up	up
	GO:0007067	mitotic nuclear division		12	28		9.2 × 10^−4^	5.6 × 10^−16^		up	up
	GO:0051301	cell division		13	29		2.4 × 10^−3^	1.8 × 10^−13^		up	up
	GO:0048285	organelle fission		12	28		1.4 × 10^−3^	1.6 × 10^−15^		up	up
	GO:0000087	M phase of mitotic cell cycle		12	28		1.1 × 10^−3^	9.0 × 10^−16^		up	up
	GO:0000279	M phase		14	34		1.2 × 10^−3^	2.5 × 10^−17^			
	GO:0022403	cell cycle phase		15	39		2.8 × 10^−3^	4.9 × 10^−19^		Up	up
DNA metabolic process	GO:0006259	DNA metabolic process		18	29		7.5 × 10^−6^	3.6 × 10^−13^		down	down
	GO:0006260	DNA replication		15	19		7.5 × 10^−9^	1.8 × 10^−11^		down	down
	GO:0006281	DNA repair		11	18		9.9 × 10^−3^	1.7 × 10^−7^		down	down
	GO:0006323	DNA packaging		12			1.3 × 10^−6^			up	
	GO:0051052	regulation of DNA metabolic process		8	9		7.5 × 10^−3^	5.9 × 10^−3^		down	down
chromosome organization	GO:0006325	chromatin organization		14			5.6 × 10^−3^			up	
	GO:0006333	chromatin assembly or disassembly		11			4.4 × 10^−5^			up	
	GO:0006334	nucleosome assembly		11			7.6 × 10^−7^			up	
	GO:0031497	chromatin assembly		11			1.1 × 10^−6^			up	
	GO:0034728	nucleosome organization		11			2.1 × 10^−6^			up	
	GO:0034622	cellular macromolecular complex assembly		15			1.2 × 10^−4^			up	
	GO:0043933	macromolecular complex subunit organization		19			5.2 × 10^−3^			up	
	GO:0051276	chromosome organization		19	22		1.9 × 10^−5^	1.6 × 10^−3^		up	up
	GO:0065003	macromolecular complex assembly		19			2.1 × 10^−3^			up	
	GO:0065004	protein-DNA complex assembly		12			8.3 × 10^−8^			up	
cellular response to stress	GO:0006974	response to DNA damage stimulus			18			1.1 × 10^−5^			down
	GO:0033554	cellular response to stress			18			4.2 × 10^−3^			down
other	GO:0007017	microtubule-based process			17			2.5 × 10^−4^			up
	GO:0007059	chromosome segregation			10			1.4 × 10^−3^			up

**Figure 5:**
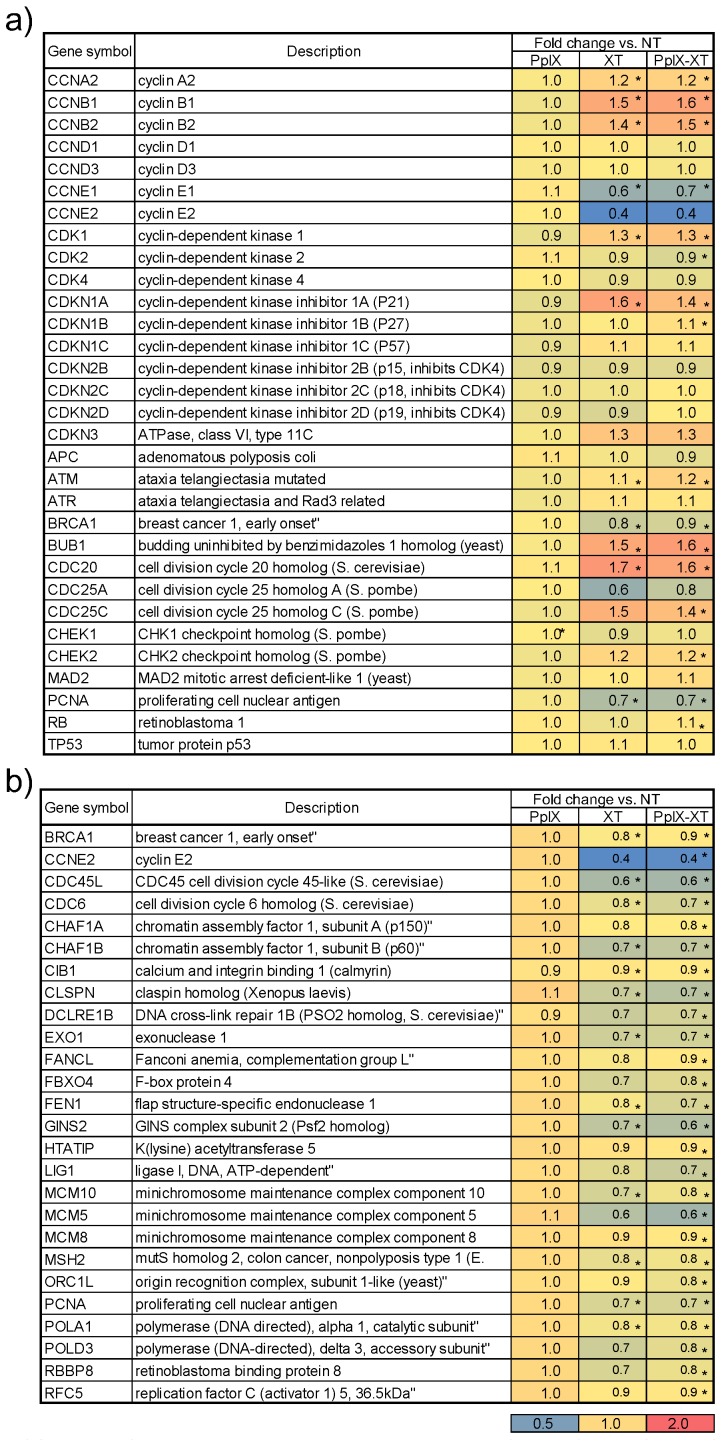
Heat map of marker genes representing (**a**) cell cycle regulation and (**b**) DNA metabolic processes. Each row represents a gene, and each column represents the average fold change in gene expression relative to the NT group (* *p* < 0.01). The colors indicate the intensity based on the log expression ratio from blue (highly negative) to red (highly positive).

We first examined marker genes involved in cell cycle regulation, because the functional analysis identified 15 GO terms related to this process. Figure 5a shows the heat map of marker gene expressions for cell cycle regulation. Each row represents a gene, and each column represents the ratio of the average fold change in gene expression relative to the NT group, as well as associated *p*-values. The color intensity depicts the degree of expression; the blue color indicates highly negative expression, while red indicates highly positive expression. Cyclins, cyclin-dependent kinases and cyclin-dependent kinase inhibitors, among other genes, were selected as markers of the cell cycle. CCNE1, CCNE2 and CDK4 were downregulated, whereas Cdkn1a was upregulated in the XT and PpIX-XT groups. Furthermore, upregulation of ATM, CHK2 and CDKN1A indicated inhibition of DNA replication. Figure 5b shows the heat map of the selected genes for the DNA metabolic process (GO:0006259). Key genes involved in DNA replication, such as PCNA, the MCM complex (MCM5, MCM8 and MCM10), LIG1 and DNA polymerase (POLA1, POLD3), were downregulated relative to the NT group. Therefore, XT and PpIX-XT treatment arrested the tumor cells at the G1 transition of the cell cycle and inhibited DNA replication, whereas PpIX treatment showed no effect on the cell cycle or on the DNA replication compared to the NO group. 

Ionizing radiation typically induces direct and/or indirect damage to DNA, triggering various cellular responses, including cell-cycle arrest, transformation and cell death [20,21,22]. The results of our study suggest a similar pattern in response to DNA damage. The PpIX-XT group differed from the XT group in terms of quantitative gene expression, but qualitative gene expression was the same in both groups.

In the case of genes selected for chromosome organization, we found that several core histones (HIST1H2BB, HIST1H2BD, HIST1H2BE, HIST1H2BH, HIST1H2BJ, HIST1H2BL, HIST1H2BO, HIST2H2BE and HIST3H2BB) were upregulated in both the XT and PpIX-XT groups. A significant amount of the histone synthesis occurs during the S phase. The synthesis of the histone proteins is tightly coupled to DNA synthesis [23,24]. Histone synthesis in cycling tissue culture cells can be separated into basal synthesis and S phase synthesis. The upregulation of histone gene expressions in this study was caused by the basal synthesis or by transitional response. The genes responsible for cellular responses to stress were identical to the genes involved in DNA metabolic processes.

In our previous *in vivo* study, treatment with 3-Gy irradiation at 10 intervals significantly suppressed tumor growth. However, in this study, we irradiated the cells with a single dose (3 Gy) following administration of PpIX. This dose appeared to be weak and ineffective at suppressing tumor growth. Therefore, we evaluated microarray data, which revealed systematic changes in cellular biological responses, as well as differences between the PpIX-XT and XT treatments.

## 4. Conclusions 

To assess the effects of PpIX administration prior to X-ray irradiation on cancer cells *in vitro*, we analyzed gene expression by microarray. We evaluated gene expression differences between HeLa cell groups treated with PpIX (PpIXT), X-ray irradiation (XT) and both treatments combined (PpIX-XT) at sub-lethal doses in cells with limited proliferation capacity. Under these conditions, we confirmed the intracellular accumulation of porphyrin, as well as ROS generation. XT and PpIX-XT induced systematic changes in the expression of genes related to cell-cycle arrest and inhibition of DNA replication. However, the changes in gene expression associated with PpIX administration alone, without X-ray irradiation, were less extensive and not systematic. Interestingly, PpIX-XT and XT differed in the number of genes that showed altered expression profiles relative to controls, but qualitative gene expression remained the same between the two groups. The increase in intracellular ROS levels after PpIX and/or X-ray treatment may have led to differences in clonogenic survival between different treatment groups, and this may have resulted from differences in gene expression between the PpIX-XT and XT groups. Our data highlight the complex mechanism by which PpIX enhances ROS generation, resulting in a decrease in clonogenic survival via DNA damage-induced cell-cycle arrest. The present study elucidates the cellular responses to PpIX, which acts as a radiosensitizer under non-lethal and limited proliferation conditions. Our findings could be useful in improving the efficacy of radiotherapy to treat human cancers.
